# Neuropeptide Receptor Transcriptome Reveals Unidentified Neuroendocrine Pathways

**DOI:** 10.1371/journal.pone.0003048

**Published:** 2008-08-25

**Authors:** Naoki Yamanaka, Sachie Yamamoto, Dušan Žitňan, Ken Watanabe, Tsuyoshi Kawada, Honoo Satake, Yu Kaneko, Kiyoshi Hiruma, Yoshiaki Tanaka, Tetsuro Shinoda, Hiroshi Kataoka

**Affiliations:** 1 Department of Integrated Biosciences, Graduate School of Frontier Sciences, The University of Tokyo, Chiba, Japan; 2 Institute of Zoology, Slovak Academy of Sciences, Bratislava, Slovakia; 3 Suntory Institute for Bioorganic Research, Osaka, Japan; 4 Faculty of Agriculture and Life Sciences, Hirosaki University, Aomori, Japan; 5 National Institute of Agrobiological Sciences, Ibaraki, Japan; University of the Western Cape, South Africa

## Abstract

Neuropeptides are an important class of molecules involved in diverse aspects of metazoan development and homeostasis. Insects are ideal model systems to investigate neuropeptide functions, and the major focus of insect neuropeptide research in the last decade has been on the identification of their receptors. Despite these vigorous efforts, receptors for some key neuropeptides in insect development such as prothoracicotropic hormone, eclosion hormone and allatotropin (AT), remain undefined. In this paper, we report the comprehensive cloning of neuropeptide G protein-coupled receptors from the silkworm, *Bombyx mori*, and systematic analyses of their expression. Based on the expression patterns of orphan receptors, we identified the long-sought receptor for AT, which is thought to stimulate juvenile hormone biosynthesis in the corpora allata (CA). Surprisingly, however, the AT receptor was not highly expressed in the CA, but instead was predominantly transcribed in the corpora cardiaca (CC), an organ adjacent to the CA. Indeed, by using a reverse-physiological approach, we purified and characterized novel allatoregulatory peptides produced in AT receptor-expressing CC cells, which may indirectly mediate AT activity on the CA. All of the above findings confirm the effectiveness of a systematic analysis of the receptor transcriptome, not only in characterizing orphan receptors, but also in identifying novel players and hidden mechanisms in important biological processes. This work illustrates how using a combinatorial approach employing bioinformatic, molecular, biochemical and physiological methods can help solve recalcitrant problems in neuropeptide research.

## Introduction

Diverse biological processes in metazoans such as development, reproduction and behavior are regulated by neuropeptides. One of the most effective approaches for analyzing their physiological functions is to identify their receptors, most of which belong to the G protein-coupled receptor (GPCR) superfamily. In insects, *Drosophila melanogaster* has been used as a model organism for such an approach. To date, some forty GPCRs are categorized as neuropeptide GPCRs in *Drosophila*, and about two-thirds of these receptors have been “deorphanized” (*i.e.* their endogenous ligands have been found) [1], [2].

In spite of this success in flies, receptors for several key neuropeptides in insect development are yet to be identified. Among such neuropeptides is allatotropin (AT), which is the only neuropeptide widely thought to stimulate juvenile hormone (JH) biosynthesis [3], [4]. JH is involved in various aspects of insect physiology such as development, reproduction and polyphenism [5]–[7], but the regulatory mechanisms that control its biosynthesis are not well understood in part due to the lack of information on several key molecules such as the AT receptor. The identities of receptors for prothoracicotropic hormone and eclosion hormone, two other important players in the regulation of insect development, also remain uncharacterized.

Considering that the target organs of these key neuropeptides are known, comprehensive tissue expression analyses of the remaining orphan receptors seems likely to be informative in identifying their receptors. With just a few exceptions, however [8], [9], such spatial expression analyses have been hampered due to the small size of the *Drosophila*. In contrast, larger lepidopteran insects such as *Manduca sexta* and *Bombyx mori* allowed detailed tissue expression analyses of several neuropeptide GPCRs, which have helped elucidate novel functions of their ligands [10]–[12]. Now that a draft sequence of the *Bombyx* genome has been published [13], [14], a comprehensive analysis of an entire neuropeptide GPCR transcriptome is feasible for the first time among lepidopterans.

Using *Bombyx*, we report here the first comprehensive cloning and thorough analyses of neuropeptide GPCRs conducted in any organism. This approach, combined with biochemical and physiological studies, led to the molecular characterization of the first AT receptor, as well as the identification of novel allatoregulatory peptides. Our results clearly show the effectiveness of the systematic analysis of a receptor transcriptome in neuropeptide research, and have enabled us to formulate a novel hypothesis for how JH biosynthesis is regulated by AT.

## Results

### Comprehensive Cloning of *Bombyx* Neuropeptide GPCR Genes

**Figure 1:**
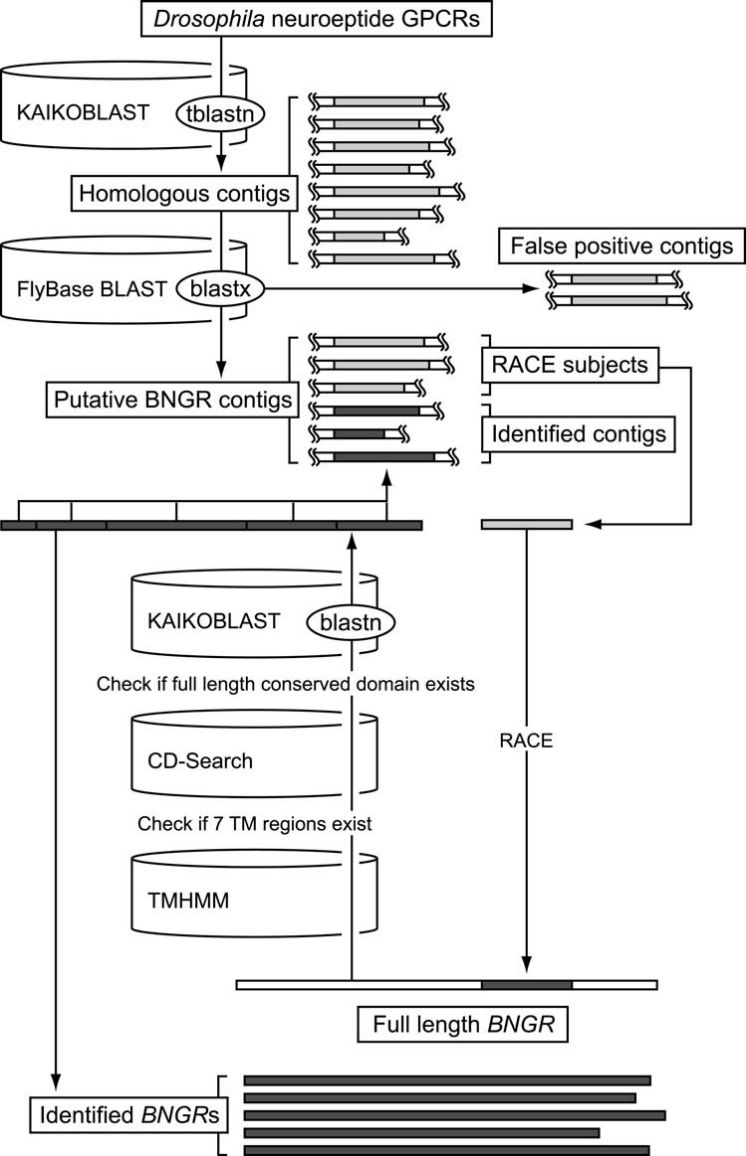
Flow chart for the comprehensive identification of *BNGR*s. Rectangles indicate nucleotide sequences. Light gray denotes homologous sequence to *Drosophila* neuropeptide receptors, while dark gray denotes identified *BNGR* sequence.

When a newly released genome is screened for a certain family of genes, homology searching using the family members from other well-characterized species is the most powerful and reliable approach [1], [2], [15]–[19]. In order to thoroughly identify neuropeptide GPCR genes encoded in *Bombyx* genome (*Bombyx* neuropeptide GPCR genes or *BNGR*s), therefore, we sought to identify all the homologs of previously categorized *D. melanogaster* neuropeptide GPCRs (see Figure 1 for the whole identification process). Based on earlier reports, 40 *Drosophila* GPCRs were listed as neuropeptide receptors (Table 1) [1], [2], [20]. Using the amino acid sequences of these *Drosophila* neuropeptide GPCRs as queries, *Bombyx* whole-genome shotgun sequence contigs were screened (TBLASTN analysis) by using KAIKOBLAST (http://kaikoblast.dna.affrc.go.jp/; first screening). This resulted in the identification of 195 contigs, whose E-values against at least one *Drosophila* neuropeptide GPCR are less than 0.1 (Table S1). Since the gene fragments in these contigs may encode other types of proteins whose sequences are similar to the neuropeptide GPCRs, all the contigs were further screened against *Drosophila* proteome (BLASTX analysis) using FlyBase BLAST (http://flybase.net/blast/; second screening). This second screening yielded 139 contigs, whose predicted amino acid sequences showed the highest similarities to one of the 40 *Drosophila* neuropeptide GPCRs (Table S1). These contigs were termed putative BNGR contigs, and were subjected to further analyses.

**Table 1:** List of *Drosophila* neuropeptide GPCRs.

CG No.	Family	Synonyms	Ligands	References
1147	A	NPFR1	NPF	[55]
2114	A	FR, FMRFaR	FMRFamide	[56], [57]
2872	A	AlstR, DAR-1	allatostatin A	[58], [59]
4395	B			
5811	A	NepYr		[60]
5911	A	ETHR	ETH	[21], [61]
5936	A	CG16958, CG33639		
6111	A	CcapR, CG14547, CG33344	CCAP	[24], [62]
6515	A	Takr86C, NKD	tachykinin	[63], [64]
6857	A	CCKLR-17D1	sulfakinin	[2]
6881	A	CCKLR-17D3, DSK-R1, CG6861, CG6894, CG32540	sulfakinin	[65]
6986	A		proctolin	[66], [67]
7285	A	star1, Drostar1	allatostatin C	[36]
7395	A	NPFR76F, Drm-sNPF-R, CG18639	short NPF	[68]
7887	A	Takr99D, DTKR	tachykinin	[64], [69]
8422	B		DH 44	[70]
8784	A		pyrokinin-2	[62], [71]
8795	A		pyrokinin-2	[62], [71]
8985	A	DmsR-1	myosuppressin	[23]
9918	A		pyrokinin-1	[72]
10001	A	AlstR2, DAR-2, AR-2	allatostatin A	[59]
10626	A	Lkr, DLKR	leucokinin	[73]
10698	A	GRHRII, DCR	corazonin	[62], [74]
10823	A		SIFamide	[29]
11325	A	GRHR, DAKHR	AKH	[27], [62]
12370	B			
12610	A	CG32547, CG15049, CG15050		
13229	A			
13575	A			
13702	A	AlCR2, Drostar2	allatostatin C	[36]
13758	B	PDFR, groom-of-PDF, Han	PDF	[75]–[77]
13803	A	DmsR-2	myosuppressin	[23]
13995	A			
14003	A	CG14002, CG31645		
14484	A	CG18192, CG30106	allatostatin B	[64]
14575	A	capaR	capa	[22], [62]
14593	A	CG14594		
16726	A	CG32047, CG33696		
17415	B	CG17043, CG32843	DH 31	[78]
30340	A	BACR48G21.1		

AKH, adipokinetic hormone; CCAP, crustacean cardioactive peptide; DH, diuretic hormone; ETH, ecdysis-triggering hormone; NPF, neuropeptide F; PDF, pigment-dispersing factor.

Considering that the number of BNGRs is likely to be similar to that of *Drosophila* neuropeptide GPCRs, the large number of putative BNGR contigs clearly suggests that most *BNGR*s are fragmented into several contigs, making it impossible to predict the whole structure of a *BNGR* from the published genome sequence. Therefore, the best and perhaps the only way to identify all the *BNGR*s from this incomplete genome information is to perform rapid amplification of cDNA ends (RACE) from each putative gene fragments. Although this is quite laborious, cloned cDNA sequences are even more reliable than the predicted ones, as evidenced by the repeated correction of *Drosophila* GPCR gene sequences every when they are actually cloned [21]–[24].

In *Bombyx*, 8 genes have been previously reported to encode neuropeptide GPCRs [11], [12], [25]–[30]. BLAST searching for the contigs containing the fragments of these genes revealed that fragments of all the 8 genes are included in 23 putative BNGR contigs, yielding an average of 2.9 putative BNGR contigs for each gene (Table S2). This ensures the comprehensiveness of the putative BNGR contig repertoire, because a single fragment of a gene is enough to determine the whole structure of the cDNA by RACE.

**Figure 2:**
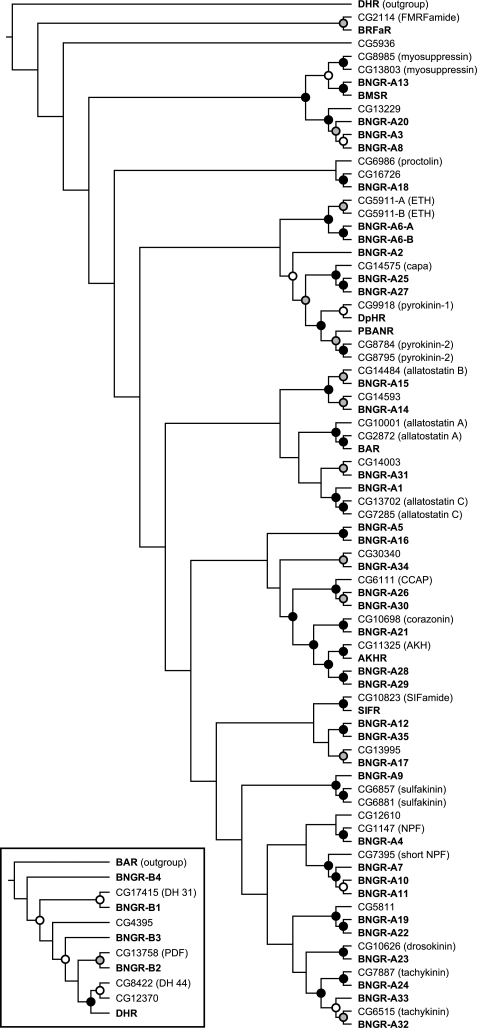
Neighbor-joining phylogenetic trees for neuropeptide GPCRs in *Bombyx* and *Drosophila*. The trees are rooted with DHR and BAR as outgroups for family A (rhodopsin-like) and family B (secretin receptor-like; shown in inset) GPCRs, respectively. Bootstrap support is indicated with an open circle for 50–70%, gray circle for 70–90%, and filled circle for 90–100%. *Bombyx* GPCRs are in bold type: AKHR, adipokinetic hormone receptor; BAR, *Bombyx* allatostatin (A-type) receptor; BMSR, Bommo-myosuppressin receptor; BRFaR, Bommo-FMRFamide receptor; DHR, diuretic hormone receptor; DpHR, diapause hormone receptor; PBANR, pheromone biosynthesis-activating neuropeptide receptor; SIFR, SIFamide receptor. The endogenous *Drosophila* ligands are given in parentheses: AKH, adipokinetic hormone; CCAP, crustacean cardioactive peptide; DH, diuretic hormone; ETH, ecdysis-triggering hormone; NPF, neuropeptide F; PDF, pigment-dispersing factor.

After removing the contigs attributed to the reported genes, RACE was applied to each of the remaining 116 contigs. Based on the obtained cDNA sequences, the whole coding region of each *BNGR* was cloned into pME18S, a mammalian expression vector. The cloned *BNGR* was sequenced, and the predicted amino acid sequence was submitted to TMHMM (http://www.cbs.dtu.dk/services/TMHMM/) [31] and CD-Search (http://www.ncbi.nlm.nih.gov/Structure/cdd/wrpsb.cgi) [32], respectively, in order to check if 7 transmembrane regions and other characteristic features of GPCRs are conserved in each individual BNGR. The certified *BNGR* coding sequence was then submitted to KAIKOBLAST (BLASTN analysis), to find all the contigs containing the fragments of the cloned *BNGR*. These identified contigs were removed from the list of putative BNGR contigs, and the next RACE was started from one of the residual contigs. This process was repeated until all the contigs were identified, which resulted in the identification of 39 novel *BNGR*s, with two splicing variants for *BNGR-A6* (Table S3). Among putative BNGR contigs, contig 227253 was shown to contain a fragment of a gene which has high similarity to mammalian melatonin receptors (*MTNR*). Contig 198123 and 280624 contained fragments of a pseudogene which has multiple stop codons within the conserved domain of GPCRs. Based on the predicted amino acid sequences, phylogenetic analysis was performed for *Bombyx* and *Drosophila* neuropeptide GPCRs (Figure 2).

### Quantitative Expression Analysis of *BNGR*s

**Figure 3:**
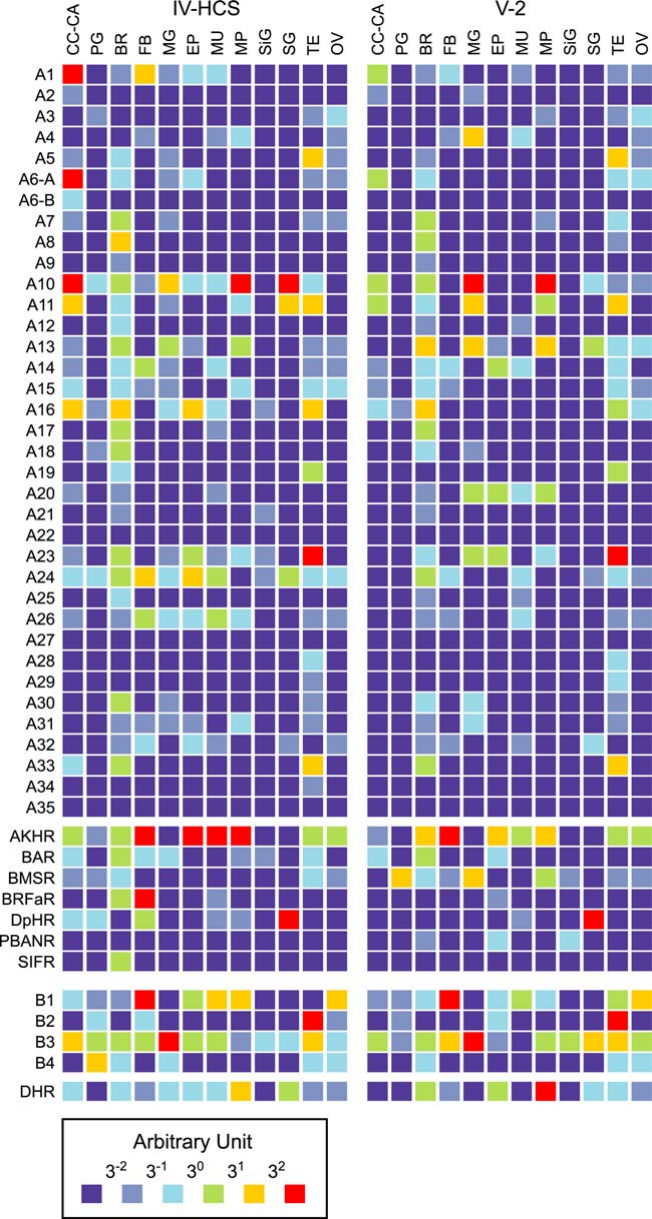
Quantitative tissue expression analyses of *Bombyx* neuropeptide GPCRs on two larval stages. Transcript levels of the GPCR genes are presented as arbitrary units. See Figure 2 for the abbreviated GPCR names. IV-HCS, fourth instar head capsule slippage; V-2, fifth instar day 2; CC-CA, corpora cardiaca-corpora allata complex; PG, prothoracic gland; BR, brain; FB, fat body; MG, midgut; EP, epidermis; MU, muscle; MP, Malpighian tubule; SiG, silk gland; SG, salivary gland; TE, testis; OV, ovary.

As a first step toward elucidating neuropeptide signaling pathways, quantitative tissue expression analysis was performed on two larval stages (Figure 3). Among the previously characterized neuropeptide GPCRs, adipokinetic hormone receptor, diuretic hormone receptor and Bommo-myosuppressin (BMS) receptor were highly expressed in fat body, Malpighian tubules and the prothoracic gland of the last instar larvae, respectively. These expression patterns correspond well to the target tissues of their ligands [11], [33], [34], suggesting that this type of expression analysis is useful for identifying regulatory circuitry. As for the BMS receptor, significant expression was observed not only in the prothoracic gland, but also in the midgut and Malpighian tubules, as shown in the previous report [11]. Indeed, the tissue expression analysis showed that many receptors are expressed in numerous tissues, suggesting multifunctional features of their ligands.

### Functional Characterization of CC-CA-Expressed Receptors

For detailed studies, we then focused on the six receptors highly expressed in the CC-CA complex: BNGR-A1, A6-A, A10, A11, A16 and B3. There have been two neuropeptides reported to regulate JH production by lepidopteran CA: allatostatin (*Manduca*-type or C-type; AST-C) and allatotropin (AT) [3], [4], [35]. In spite of the pivotal role of JH in numerous physiological processes, functions of these regulatory peptides have not been sufficiently analyzed, in part due to the lack of information of their receptors [4].

**Figure 4:**
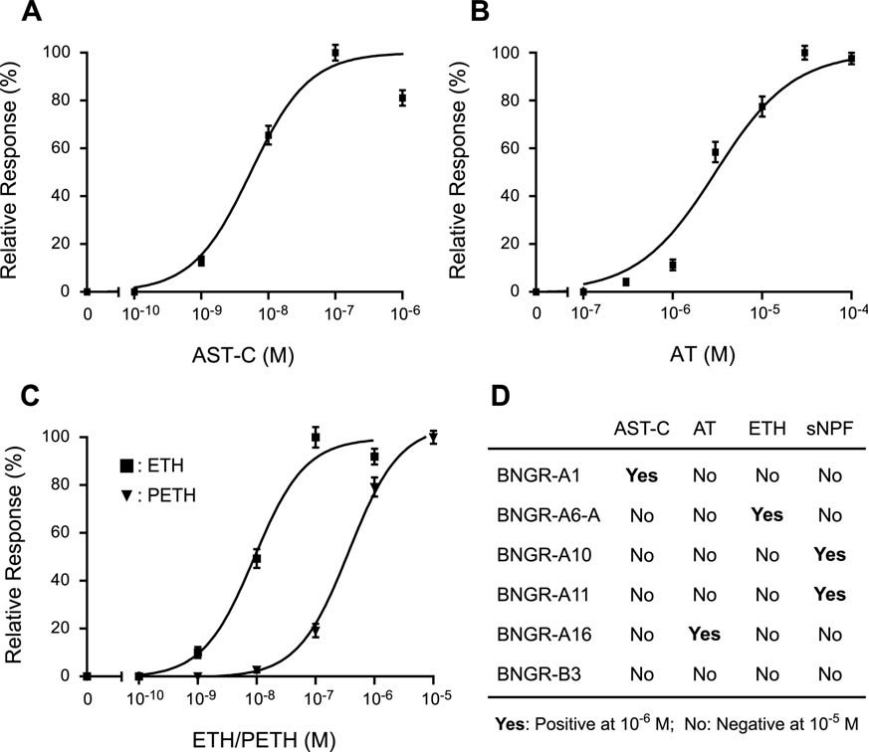
Functional characterization of CC-CA-expressed receptors. (A–C) Ca^2+^ imaging analysis using HEK293 cells expressing BNGR-A1 (A), BNGR-A16 (B) and BNGR-A6-A (C). Dose-response curves for AST-C (A), AT (B), ETH and pre- ecdysis-triggering hormone (PETH; C) are shown. Vertical bars represent S.E. Each datum point was calculated from the responses of 123-165 cells in three independent experiments. (D) Summary of the ligands for the CC-CA-expressed BNGRs analyzed by Ca^2+^ imaging analysis.

To begin a characterization of these six BNGRs, each was functionally analyzed using a heterologous expression system. HEK293 cells expressing BNGR-A1 responded to AST-C in a dose-dependent manner (Figure 4A). This result is reasonable, since the *Drosophila* homologs of BNGR-A1 (CG7285 and CG13702; see Figure 2) have been identified as receptors for AST-C-like peptide [36]. Moreover, BNGR-A16 responded to AT specifically and dose-dependently (Figure 4B), showing that this receptor is the functional AT receptor. No clear homolog of *BNGR-A16* was found in the *Drosophila* genome as expected (Figure 2), since there is no AT neuropeptide gene in the fly [1], [37], [38]. The identification of the long-sought AT receptor clearly shows the efficiency of our approach in identifying unknown ligands for orphan receptors.

### Reverse-Physiological Identification of Novel Allatostatic Peptides

Another advantage of using *Bombyx* is that biochemical approaches like peptide purification from organ extracts and physiological techniques like organ culture can be combined with functional analyses of the receptors. We therefore sought to purify unknown ligands for the rest of BNGRs expressed in the CC-CA complex, in pursuit of other regulators of JH biosynthesis. Based on the homology with *Drosophila* GPCRs, BNGR-A6-A was revealed to be the receptor for ecdysis-triggering hormone (ETH; Figure 4C), while BNGR-A10 and A11 are likely the receptors for short neuropeptide F (sNPF). ETH, which is produced in Inka cells of epitracheal glands and released into the hemolymph during ecdysis, is one of the best-characterized peptides in insects [9], [39]. On the other hand, the physiological functions of sNPF are not well characterized [37], [40], [41], and *Bombyx* sNPF remains to be identified.

**Figure 5:**
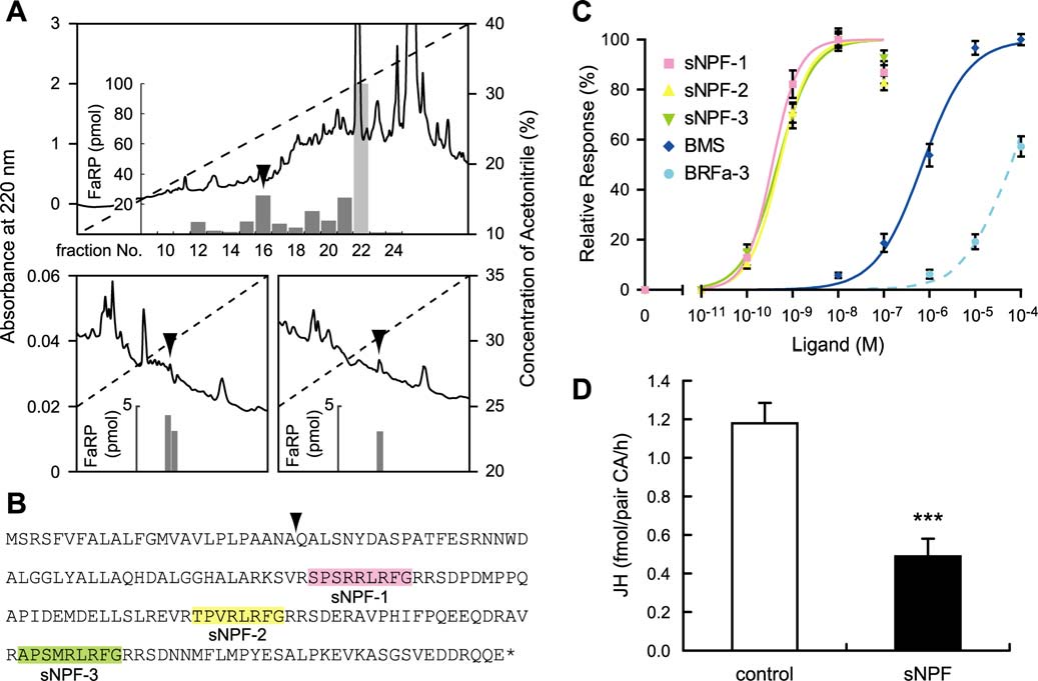
Reverse-physiological identification of sNPFs as novel allatostatic peptides. (A) Purification of sNPFs (arrowheads). The first (upper panel) and the fourth (lower two panels) HPLC separation steps are shown. Solid lines denote UV absorbance, while dashed lines denote the acetonitrile gradient. Fraction No. 22 in the upper panel was originally subjected to purification of BMS, and FMRFamide-related peptide (FaRP) content in this fraction was presented as the amount of purified BMS. (B) The deduced amino acid sequence of the sNPF precursor. The putative site of the signal peptide cleavage is indicated with an arrowhead, and the mature peptides are highlighted. For each mature peptide, C-terminal glycine residue is necessary for amidation. (C) Ca^2+^ imaging analysis using HEK293 cells expressing BNGR-A10. Relative response was expressed as a percentage of the highest response (induced by 10^−8^ M sNPF-1–3). For each ligand, each datum point was calculated from the responses of 80–122 cells in three independent experiments. (D) The effect of sNPF mixture (1 µM each) on JH biosynthesis by the fifth instar day 0 CA. Vertical bars represent S.E. n = 6 repeats. ***P<0.001 from Student's t-test.

Therefore we purified two sNPF peptides in *Bombyx* from brain extracts, originally prepared for the purification of BMS and Bommo-FMRFamide (BRFa) [11], [12], using an ELISA system. Four HPLC purification steps were required to purify two sNPF peptides into homogeneity, sufficient for MALDI-TOF MS analysis (Figure 5A). Aliquots of the isolated fractions were subjected to MALDI-TOF MS, which yielded the sequences TPVRLRFamide and SMRLRFamide (Figure S1). Based on these amino acid sequences, the cDNA encoding the *Bombyx* sNPF precursor was obtained and three putative mature peptides were found in the precursor sequence (Figure 5B; the purified SMRLRFamide seems to be the truncated form of sNPF-3). Functional analyses of BNGR-A10 and A11 revealed that these are indeed the sNPF receptors (Figure 4D and Figure 5C). Although these receptors also responded to BMS and BRFa, their sensitivity to sNPFs was more than 1,000 times higher than that to BMS and BRFa (Figure 5C and data not shown).

We then investigated the putative regulatory role of sNPFs in JH biosynthesis using *in vitro* CA culture. Addition of sNPFs to the incubation medium resulted in clear inhibition of JH biosynthesis (Figure 5D), suggesting they likely have allatostatic activity *in vivo*. The identification of these novel allatostatins again demonstrates the effectiveness of our approach in the investigation of unknown neuropeptide functions.

### Identification of Unexpected Signaling Network within CC-CA Complex

**Figure 6:**
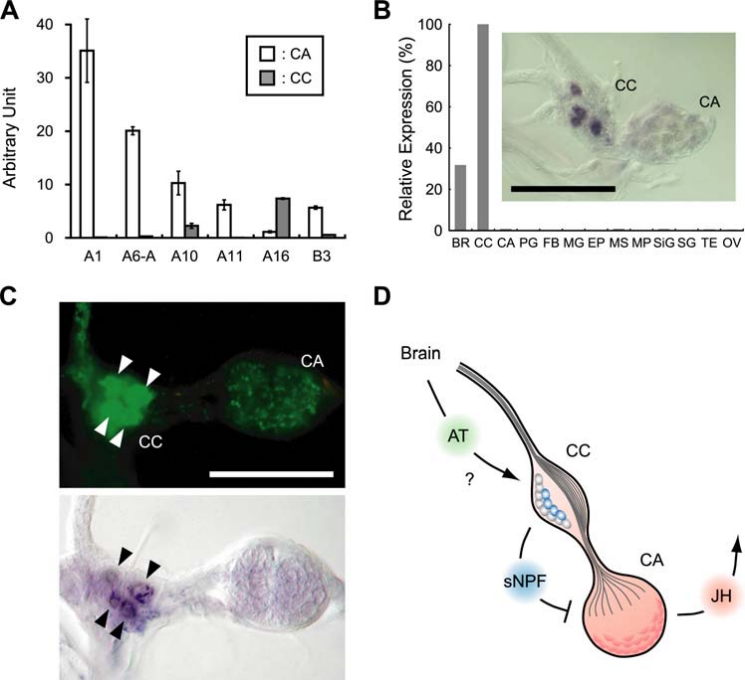
Expression of the AT receptor in sNPF-producing CC cells. (A) Expression analysis of 6 BNGRs within CC-CA complex on the fourth instar head capsule slippage. Vertical bars represent S.E. n = 3. Arbitrary unit is the same to Figure 3. (B) Expression analysis of *sNPF* on the fourth instar head capsule slippage. Relative expression was shown as a percentage of the expression level in the CC. For tissue names, see Figure 3. Inset, *in situ* hybridization of *sNPF*. Scale bar, 100 µm. (C) Double staining of the CC-CA with immunohistochemistry for sNPF (upper) and *in situ* hybridization for *BNGR-A16* (lower). Scale bar, 100 µm. (D) Schematic illustration of the regulatory pathways of JH biosynthesis identified in this study. Putative role of AT in the regulation of sNPF production in the CC remains to be elucidated.

Surprisingly, in the course of detailed expression analysis of the six *BNGR*s, we found that *BNGR-A16* is mainly expressed in the CC, not in the CA (Figure 6A). Considering that many previous studies on AT activity *in vitro* were conducted using the CC-CA complex, it is of interest to hypothesize that the CC affects JH biosynthesis in the CA, and AT regulates JH production indirectly via the CC. The CC is well known as the source of a peptide hormone, adipokinetic hormone [42], so it is plausible that the CC is producing other peptide ligands for the BNGRs expressed in the CA. Remarkably, RT-PCR and *in situ* hybridization analyses of *sNPF* revealed its expression mainly in the CC (Figure 6B). This result was also confirmed by direct MS analysis, which showed the existence of all three mature sNPF peptides in the CC-CA (Figure S2). Finally, double staining using immunohistochemistry and *in situ* hybridization revealed AT receptor expression in the sNPF-producing cells in the CC (Figure 6C). These unexpected findings suggest that AT regulates the production and/or the release of sNPFs from the CC, and at least some of the AT functions on JH biosynthesis may be mediated by sNPF (Figure 6D).

## Discussion

In this study, we conducted a comprehensive cloning and the systematic expression analysis of neuropeptide GPCRs in the silkworm *Bombyx mori*. These analyses led to the following major findings: 1) identification and functional characterization of the first AT receptor, 2) purification and identification of novel allatostatic peptides produced in the CC, and 3) elucidation of an unexpected signaling network within CC-CA complex, potentially mediated by the above two factors.

Our finding that the AT receptor is predominantly expressed not in the CA but in the sNPF-producing cells of the CC raises an intriguing possibility that the allatotropic activity of AT may be mediated by the CC (Figure 6D). In support of this idea, previous assays for AT activity have been mostly done with CC-CA complexes, rather than the CA alone. If AT indeed has any effect on the production and/or the release of sNPF from the CC, such effect is most likely inhibitory, because sNPFs have clear allatostatic activity (Figure 5D). Thus, AT could inhibit the allatostatic effect of sNPFs produced in the CC, thereby exerting its indirect allatotropic effect by “derepression”. Our preliminary results that the larval CA incubated alone secretes more JH than CC-CA complex *in vitro* (Y.K. and K.H., unpublished) further support this idea.

However, this hypothesis must be carefully considered from several aspects. First, some allatoregulatory peptides are known to change their activity during development [43]. Detailed physiological analyses of sNPF and AT along developmental stages, as well as the improvement of a reliable *in vitro* CC-CA co-culture system, are thus necessary to examine the above hypothesis. Second, we cannot fully rule out the possibility that the low, but still detectable expression of the AT receptor in the CA (Figure 6A) may mediate the allatotropic activity of its ligand, though we could not detect any allatotropic activity of AT using our *in vitro* CA culture (data not shown). Considering the varying activity of AT during development in many insects [4], [44], the temporal expression analysis of the AT receptor within the CC-CA complex, as well as the other receptors expressed in the CA, may help clarify such a possibility. Thus, both of these alternative possibilities require the introduction of the “fourth”, temporal axis to our three-dimensional, spatial expression analysis. Although this is beyond the scope of this study, our work provides a firm foundation upon which such further analyses can be based.

Considering that sNPF has been reported in most insects examined [37], it is possible that sNPF has a conserved role in the regulation of JH biosynthesis. In relation to this, it is interesting to note that several physiological events related to sNPF in other insects (ovarian growth, body size control and adult diapause) are all reminiscent of its regulatory role in JH biosynthesis [37], [40], [41], [45]. Together with the recently reported role of sNPF in the regulation of insulin expression [46], investigation of the functional conservation of sNPF in other insect species is clearly warranted.

Identification of sNPFs as CC-produced allatoregulatory peptides may also provide a direct link between JH biosynthesis and energy balance. JH acts as a signal for starvation-induced growth inhibition, which suggests a tight link between JH biosynthesis and energy balance [47]. On the other hand, CC is well known for its glucose-sensing capacity, which regulates energy homeostasis in insects [42]. Investigation of the sNPF secretion from the CC under a variety of physiological conditions will test the validity of this possibility.

The high expression of the ETH receptor in the CA (Figure 6A) is another unexpected finding of this study. This result was further verified by *in situ* hybridization (Figure S3), suggesting an unknown physiological role of ETH in the regulation of JH biosynthesis. ETH is produced by Inka cells of epitracheal glands and released into the hemolymph during ecdysis [9], [39], so the putative allatoregulatory role of ETH may explain the transient rise of JH titer at ecdysis reported in many insects [48]–[50]. Together with the as-yet-unknown ligand of BNGR-B3, elucidation of the physiological significance of these putative regulators of JH biosynthesis will elucidate a number of unexpected features of this important endocrine pathway.

All of the above results clearly suggest that the regulatory mechanisms of JH biosynthesis are as complex as those of ecdysteroidogenesis, in which multiple factors are involved [51]. Indeed, most tissues investigated in this study express various neuropeptide GPCRs (Figure 3), indicating the necessity of a systematic approach to reveal regulatory mechanisms of their functions. The present study provides a solid basis for such an approach, which leads to our thorough understanding of the neuropeptide circuitry that extends beyond individual tissues to form a system-wide network in multicellular organisms.

## Materials and Methods

### Comprehensive identification of *BNGR*s

BNGR genes were identified by a cloning-based approach combining homology searching and RACE, using the SMART RACE cDNA Amplification Kit (Clontech Laboratories, Mountain View, CA) according to the manufacturer's instructions. Whole coding regions of all the identified BNGR genes were cloned, sequenced, and confirmed to have seven transmembrane regions and full conserved domains for GPCRs. See Results and Tables S1, S2, S3 for the detailed procedure. The primers used for RACE and cloning are listed in Table S4 and S5, respectively.

### Phylogenetic analysis

Deduced amino acid sequences of *Bombyx* and *Drosophila* neuropeptide receptors were aligned and rooted neighbor-joining trees were generated using ClustalW. Bootstrap analyses of 1,000 replications were conducted to assess the relationships.

### Quantitative RT-PCR (Q-RT-PCR) analysis of the neuropeptide GPCR genes

cDNAs from various tissues were synthesized and Q-RT-PCR was carried out essentially as described [52]. After PCR, the absence of unwanted byproducts was confirmed by automated melting curve analysis. Serial dilutions of pME18S plasmids containing coding sequences of the neuropeptide GPCR genes were used for standards. *Rp49* was chosen as a reference gene, and serial dilutions of a pCR2.1 plasmid containing 340 bp of *Rp49* cDNA were used for standards. The molar amounts of the GPCR genes and *Rp49* were calculated, and the transcript levels of the GPCR genes were normalized with *Rp49* levels in the same samples and presented as arbitrary units. The primers used for Q-RT-PCR are listed in Table S6.

### Cloning of *AST-C*

Using the amino acid sequence of *Manduca sexta* allatostatin (GenBank accession no. P42559) as a query, BLAST analysis (TBLASTN) was performed against *Bombyx* whole-genome shotgun sequence contigs using KAIKOBLAST. Based on the putative coding sequence of the obtained contig (contig 454046, GenBank accession no. BAAB01095856), RACE primers were designed as follows: sense primer, 5′-ATGGCAGATTGGTGCGACCGTGGAGGG-3′; sense nested primer, 5′-CCGGCAGTGCTATTTCAATCCCATCTCC-3′; antisense primer, 5′-ATAGGGCGAAAGCCGGGGTAAAAGTCG-3′; antisense nested primer, 5′-GGGTAAAAGTCGTGTTATTCACTTGCGG-3′. To obtain the whole cDNA sequence of *AST-C* (GenBank accession no. AB330418), RACE was performed by using the above primers and the SMART RACE cDNA Amplification Kit. First-strand cDNA prepared from larval brain total RNA was used as a template.

### Purification of sNPF-2 and 3

Residual fractions of the first purification step of *Bombyx* pupal brain extracts, which were previously used for the purification of BMS [11], were applied to the FMRFamide ELISA as previously described [12]. One of the immunoreactive fractions (No. 16 in Figure 5A) was loaded onto a PEGASIL-300 C4P column (4.6×250 mm, Senshu Kagaku Co. Ltd., Tokyo, Japan) using a Waters 2695 Separations Module (Waters, Milford, MA). Elution was performed with a linear gradient of 10–40% acetonitrile in 0.1% trifluoroacetic acid (TFA) over 60 min at the flow rate of 1 ml/min. This resulted in the detection of two FMRFamide-positive fractions, each of which was then applied onto a PEGASIL-300 ODS-II column (4.6×250 mm, Senshu Kagaku Co. Ltd.). The column was eluted with a linear gradient of 10–40% acetonitrile in 0.1% TFA over 60 min at a flow rate of 0.5 ml/min. Each immunoreactive fraction was further purified by passing over a PEGASIL-300 ODS-II column (4.6×250 mm) eluting it with a linear gradient of 10–40% acetonitrile in 0.1% heptafluorobutyric acid over 60 min at the flow rate of 0.5 ml/min.

### MALDI-TOF MS

Mass spectra and postsource decay (PSD) mass spectra of peptides were obtained with a AXIMA-CFR (Shimadzu Corporation, Kyoto, Japan) mass spectrometer. Re-crystallized α-cyano-4-hydroxycinnamic acid (α-CHCA) was used as the matrix with a concentration of 5 mg/ml of acetonitrile/water (1∶1, by volume) containing 0.1% TFA. All spectra were measured in reflector mode.

### Cloning of *sNPF*

Using the amino acid sequence of one of the purified peptide [TPVR(L/I)RFG] as a query, BLAST analysis (TBLASTN) was performed against *Bombyx* whole-genome shotgun sequence contigs using KAIKOBLAST. Based on the putative coding sequence of the obtained contig (contig 432136, GenBank accession no. BAAB01085626), 5′-RACE was performed using the following primer: antisense primer, 5′-CGGAGAGAGAGCAGCTCATCCATCTCG-3′. Based on the obtained sequence of the 5′-RACE product, 3′-RACE was further performed using the following two primers: sense primer, 5′-GATCGCCATCCCGACGCCTGCGTTTCG-3′; sense nested primer, 5′-TGCGTTTCGGCCGCCGATCAGATCCCG-3′. First-strand cDNA prepared from larval brain total RNA and the SMART RACE cDNA Amplification Kit were used.

### Q-RT-PCR analysis of *sNPF*

Q-RT-PCR of *sNPF* (GenBank accession no. AB330419) was performed as described above for the neuropeptide GPCR genes. Specific primers used are as follows: sense primer, 5′-AAGTCTGTTCGATCGCCATCCCGAC-3′; antisense primer, 5′-GAGAGAGAGCAGCTCATCCATCTCG-3′. The transcript levels of the *sNPF* were normalized with *Rp49* levels in the same samples.

### *In situ* hybridization and immunohistochemistry

*In situ* hybridization and immunohistochemistry was performed as previously described [9], [12]. Specific primers used for the production of a digoxigenin-labeled probe were as follows: *sNPF* sense primer, 5′-GGAGGGCTCTATGCTCTTCT-3′; *sNPF* antisense primer, 5′-TACATCTAGGTTGCGACGAG-3′; *BNGR-A16* sense primer 1, 5′-GAGAATGAAACCTGCGTTGGAGATCC-3′; *BNGR-A16* sense primer 2, 5′-ACGCTGACTTTCATCTCTGTGGACCG-3′; *BNGR-A16* antisense primer 1, 5′-TGGTATGTCCTGGCTTGAAGGATGCC-3′; *BNGR-A16* antisense primer 2, 5′-ACGCAGTTGTGTCAGTTCTGCAGTGG-3′; *BNGR-A6-A* sense primer 1, 5′-CGCGAGAGCTAGAAAACAGG-3′; *BNGR-A6-A* sense primer 2, 5′-CCGTCGTGAATCAATGTCTG-3′; *BNGR-A6-A* antisense primer 1, 5′-TCTTTCCGAATCAACGCTCT-3′; *BNGR-A6-A* antisense primer 2, 5′-GCCACAACGGAATCAACTTT-3′. For sNPF immunohistochemistry, rabbit polyclonal antiserum to RFamide [12] was used.

### Direct MS analysis of CC-CA

CC-CA complex was dissected in sterile saline [0.85% NaCl (w/v)], and direct MS analysis was performed as previously described [12].

### Synthetic peptides

AST-C, AT, BMS, BRFa-3 and sNPF peptides were all custom synthesized and purified by HPLC. Synthetic AST-C was converted to the disulfide form by iodine oxidation and further purified by HPLC.

### HEK293 cell expression and Ca^2+^ imaging analysis of BNGRs

*BNGR*s cloned into pME18S vector were transfected into HEK293 cells with the promiscuous G protein Gα15 as described [53]. Ca^2+^ imaging analysis was performed as reported [53]. Briefly, the transfected cells were cultured for 24 h, and loaded with 2.5 µM fura-2 AM (Invitrogen, Carlsbad, CA) for 30 min at 37°C. Neuropeptides were applied sequentially to the cells for 20 s with a peristaltic pump at a flow rate of 1.5 ml/min. Fluorescence at 510 nm (excitation at 340 or 380 nm with a xenon lamp) was monitored with an intensified CCD camera. An Argus-HiSCA calcium-imaging system (Hamamatsu Photonics, Shizuoka, Japan) was used to determine the fluorescence ratio at 340 and 380 nm.

### *In vitro* radiochemical assay for JH biosynthesis

JH biosynthesis by the CA was measured using an *in vitro* radiochemical assay as previously described [54], except that the CA was used instead of the CC-CA complex. The mixture of three sNPFs (1 µM each) was added to the incubation medium.

## Supporting Information

Table S1 Summary of the two-way screening of *Bombyx* whole-genome shotgun sequence contigs. Query *Drosophila* GPCRs are aligned at the top, and the 195 contigs obtained through the first screening were listed on the left. Figures in the table indicate the BLAST scores of the first screening. Right two columns show the result of the second screening, which extracted 139 putative BNGR contigs (red).
(0.06 MB XLS)

Table S2 List of contigs containing the fragments of previously reported *Bombyx* neuropeptide GPCR genes. Putative BNGR contigs are in red letters.
(0.02 MB XLS)

Table S3 List of the newly-identified GPCRs and their corresponding contigs. Putative BNGR contigs are in red letters. Tissue sources for the cDNA clones are indicated on the right: FB, fat body; BR, brain; MG, midgut; OV, ovary; TE, testis; PG, prothoracic gland.
(0.02 MB XLS)

Table S4 List of the primers used for the RACE of *BNGR*s.
(0.03 MB XLS)

Table S5 List of the primers used for cloning of *BNGR*s. Each primer contains a restriction site indicated in the primer name, while all the sense primers incorporate Kozak sequence (“GCCACC”).
(0.02 MB XLS)

Table S6 List of the primers used for the Q-RT-PCR analysis.
(0.03 MB XLS)

Figure S1 Postsource decay mass spectra of TPVRLRFamide (A) and SMRLRFamide (B). For each peptide, upper panel is the purified sample and the lower panel is the synthetic sample. The b- and y-type ion signals are indicated at the top. Signals of the indicated mass range are magnified 10 times to facilitate comparison. The final confirmation of the leucine residue was carried out based on the cDNA sequence.
(0.39 MB TIF)

Figure S2 Direct MS analysis of CC-CA complex. The identified peaks in (A) are summarized in (B). Asterisks in (A) denote sodium adducts for the peptides 5, 9, 12 and 13.
(0.97 MB TIF)

Figure S3 *In situ* hybridization analysis of BNGR-A6-A on the CC-CA. Scale bar, 100 micrometer.
(7.87 MB TIF)
