# Supplementary figures and images for: Neuropeptide Receptor Transcriptome Reveals Unidentified Neuroendocrine Pathways
Source: PLoS One. 2008 Aug 25;3(8):e3048. doi: 10.1371/journal.pone.0003048 (PMC2516173; doi:10.1371/journal.pone.0003048)

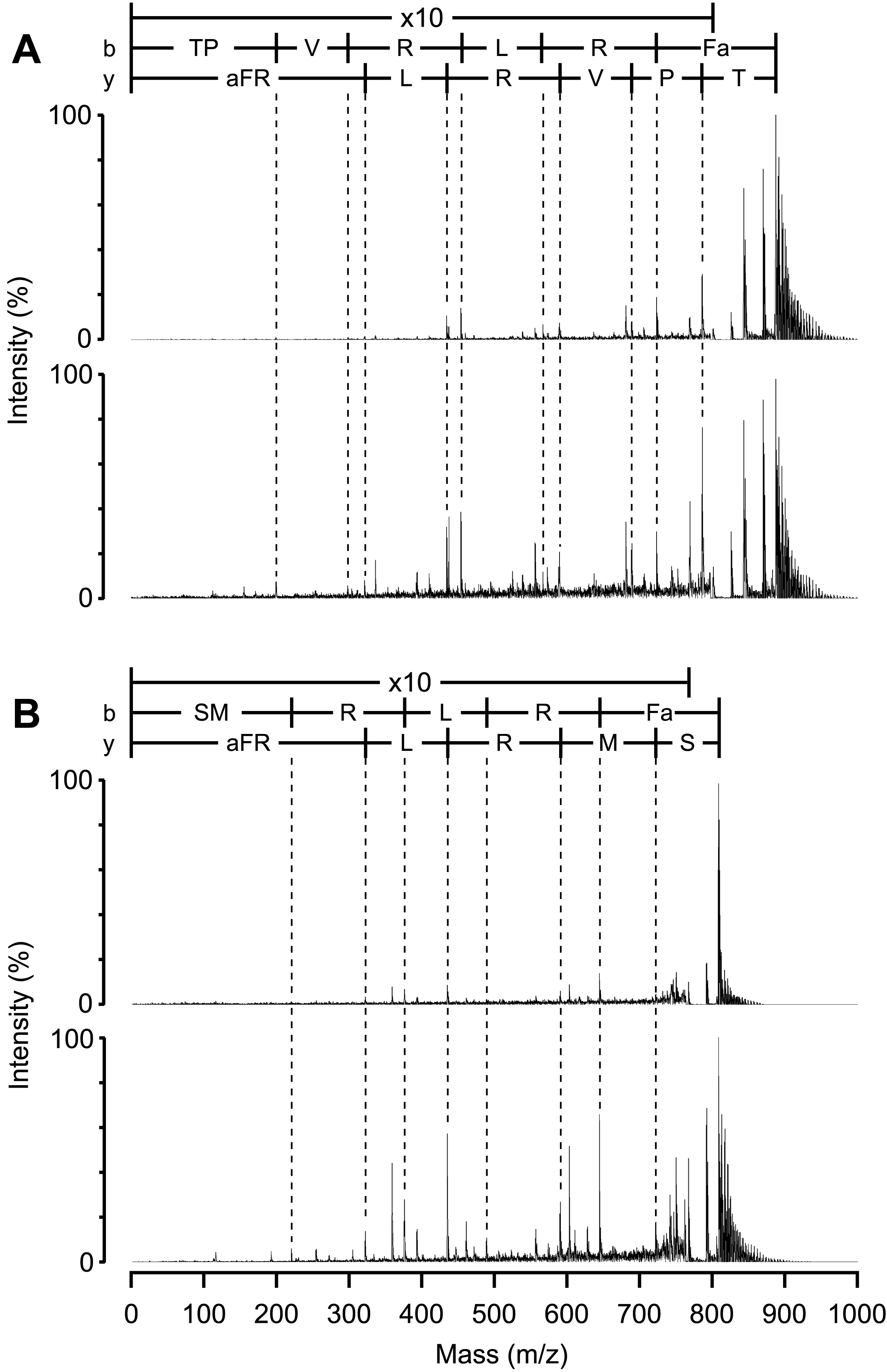

Supplement: Figure S1 — Postsource decay mass spectra of TPVRLRFamide (A) and SMRLRFamide (B). For each peptide, upper panel is the purified sample and the lower panel is the synthetic sample. The b- and y-type ion signals are indicated at the top. Signals of the indicated mass range are magnified 10 times to facilitate comparison. The final confirmation of the leucine residue was carried out based on the cDNA sequence. (0.39 MB TIF) [file pone.0003048.s007.tif]

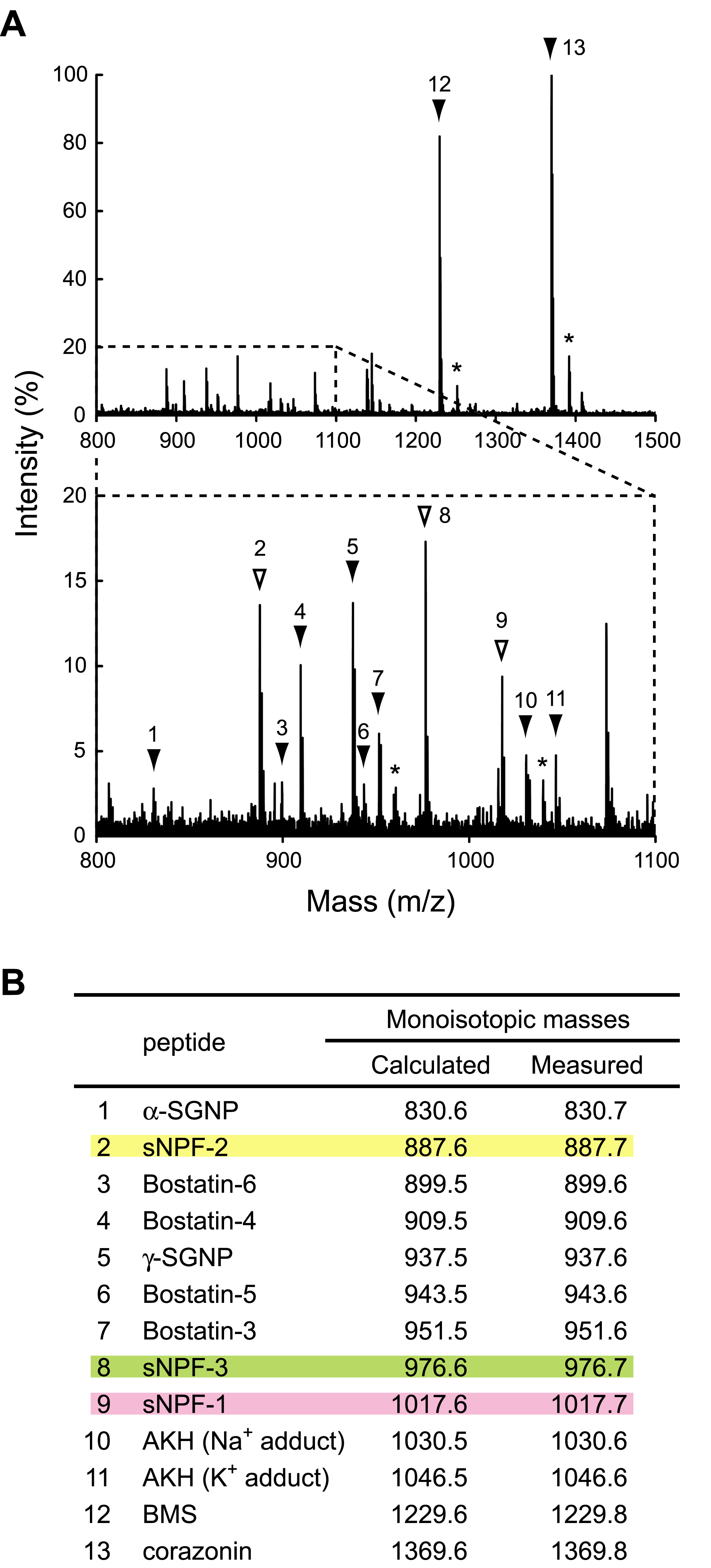

Supplement: Figure S2 — Direct MS analysis of CC-CA complex. The identified peaks in (A) are summarized in (B). Asterisks in (A) denote sodium adducts for the peptides 5, 9, 12 and 13. (0.97 MB TIF) [file pone.0003048.s008.tif]

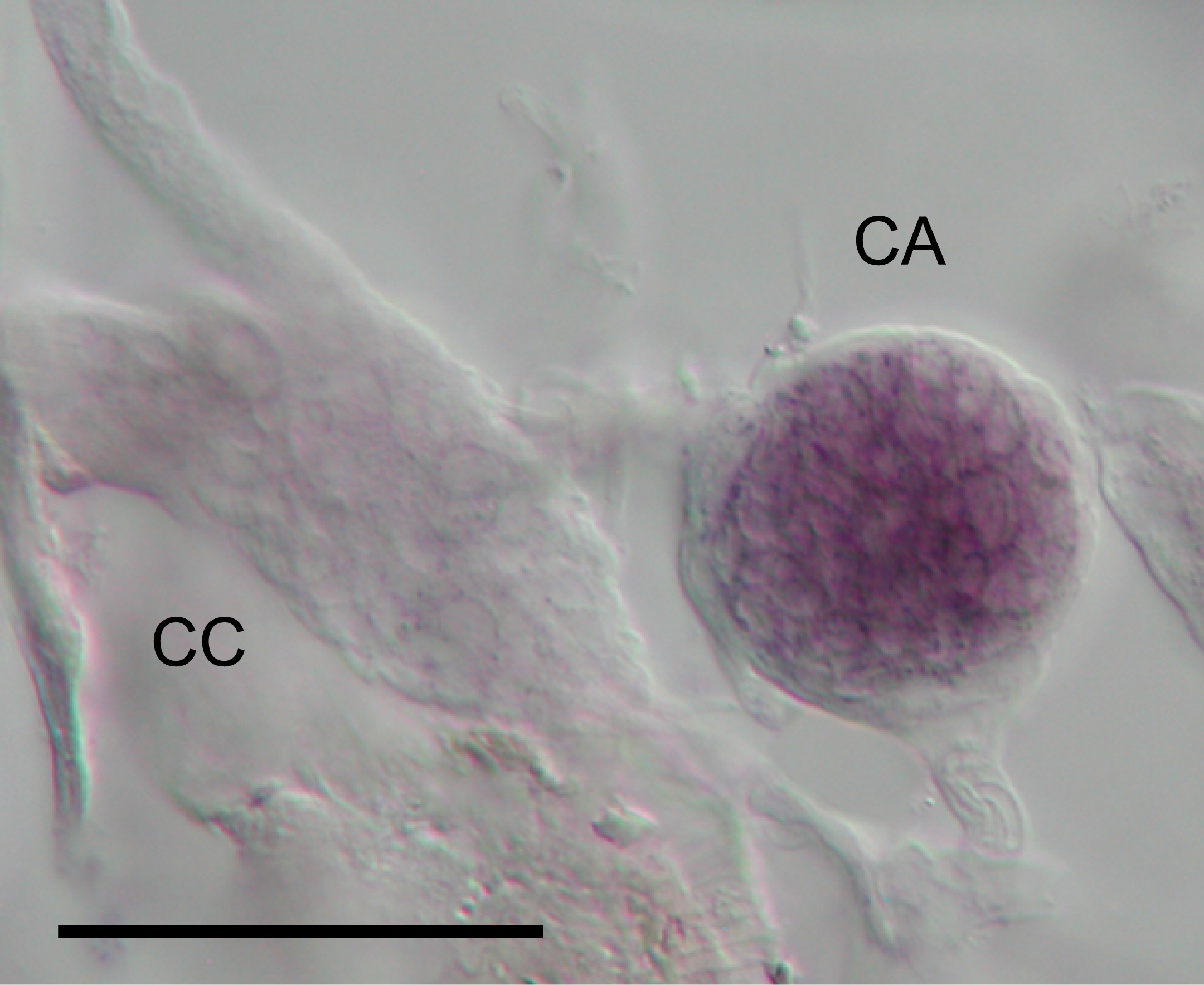

Supplement: Figure S3 — In situ hybridization analysis of BNGR-A6-A on the CC-CA. Scale bar, 100 micrometer. (7.87 MB TIF) [file pone.0003048.s009.tif]
